# Retraction of: LINC00265 targets miR-382-5p to regulate SAT1, VAV3 and angiogenesis in osteosarcoma

**DOI:** 10.18632/aging.206195

**Published:** 2025-01-31

**Authors:** Ying Xiao, Chunling Li, Hongyue Wang, Yijun Liu

**Affiliations:** 1 Department of Operating Center, The First Hospital of Jilin University, Changchun 130000, Jilin, China; 2 Department of Nephrology, The First Hospital of Jilin University, Changchun 130000, Jilin, China; 3 Department of Orthopaedics, The First Hospital of Jilin University, Changchun 130000, Jilin, China

**Keywords:** angiogenesis, vasculogenic mimicry, LINC00265, miR-382-5p, spermidine/spermine N1-acetyltransferase-1

**This article has been retracted:** Aging has completed its investigation of this paper. Several concerns were raised about the paper, including internal duplications and overlap with unrelated papers from different institutions. We found that three IHC panels from **Figure 2N** (human tumor specimens) were reproduced in **Figure 8H** (mice tumor specimens), while a panel in** Figure 5F** duplicates **Figure 6F**. In addition, images of GAPDH western blots from **Figures 5C, 7E** and** 8E**, illustrating different experiments, are identical and were also found in unrelated papers published earlier [1, 2, 3]. Article [3] also shares a transwell assay image with a panel in **Figure 5G** and transwell assay images from **Figures 5G, 6G** and** 7J** are present in an earlier paper [4]. Western blot images in **Figures 5C** and **6C** show bands duplicating those from an earlier unrelated article [5], and the blot in **Figure 8F** was found in Figure 5E of another earlier article [6]. Finally, the plate images of colony formation experiments in **Figures 5E, 6E** and** 7J** were found in multiple earlier publications [7–11], some of which have already been retracted. The Scientific Integrity office at Aging contacted the authors multiple times, but the authors did not respond to requests to clarify. The corresponding author recently stated that “our work has serious problems that need to be addressed,” but the Scientific Integrity office did not receive an official retraction letter with the signatures of all authors. In light of all these facts the Editorial decision was made to retract this paper. The Scientific Integrity office also notified the authors’ Institutions about this retraction and added their names to an Editorial Warning list.
